# Endoxifen’s Molecular Mechanisms of Action Are Concentration Dependent and Different than That of Other Anti-Estrogens

**DOI:** 10.1371/journal.pone.0054613

**Published:** 2013-01-28

**Authors:** John R. Hawse, Malayannan Subramaniam, Muzaffer Cicek, Xianglin Wu, Anne Gingery, Sarah B. Grygo, Zhifu Sun, Kevin S. Pitel, Wilma L. Lingle, Matthew P. Goetz, James N. Ingle, Thomas C. Spelsberg

**Affiliations:** 1 Department of Biochemistry and Molecular Biology, Mayo Clinic, Rochester, Minnesota, United States of America; 2 Bioinformatics Core, Division of Biomedical Statistics and Informatics, Mayo Clinic, Rochester, Minnesota, United States of America; 3 Department of Laboratory Medicine and Pathology, Mayo Clinic, Rochester, Minnesota, United States of America; 4 Department of Oncology, Mayo Clinic, Rochester, Minnesota, United States of America; Nihon University School of Medicine, Japan

## Abstract

Endoxifen, a cytochrome P450 mediated tamoxifen metabolite, is being developed as a drug for the treatment of estrogen receptor (ER) positive breast cancer. Endoxifen is known to be a potent anti-estrogen and its mechanisms of action are still being elucidated. Here, we demonstrate that endoxifen-mediated recruitment of ERα to known target genes differs from that of 4-hydroxy-tamoxifen (4HT) and ICI-182,780 (ICI). Global gene expression profiling of MCF7 cells revealed substantial differences in the transcriptome following treatment with 4HT, endoxifen and ICI, both in the presence and absence of estrogen. Alterations in endoxifen concentrations also dramatically altered the gene expression profiles of MCF7 cells, even in the presence of clinically relevant concentrations of tamoxifen and its metabolites, 4HT and N-desmethyl-tamoxifen (NDT). Pathway analysis of differentially regulated genes revealed substantial differences related to endoxifen concentrations including significant induction of cell cycle arrest and markers of apoptosis following treatment with high, but not low, concentrations of endoxifen. Taken together, these data demonstrate that endoxifen’s mechanism of action is different from that of 4HT and ICI and provide mechanistic insight into the potential importance of endoxifen in the suppression of breast cancer growth and progression.

## Introduction

Tamoxifen is a selective estrogen receptor modulator (SERM) that is commonly used for the treatment of women with endocrine responsive breast cancer. The majority of these individuals eventually develop resistance to this drug [1] and 30–50% of patients subsequently die of their disease [2], [3]. Although years of research have sought to understand the basis for this disparity in patient outcome, the mechanisms underlying this phenomenon remain poorly understood.

Tamoxifen, like many therapeutic agents, is a pro-drug that is extensively metabolized in humans by the cytochrome P450 enzyme system into 4-hydroxy-tamoxifen (4HT) and N-desmethyl-tamoxifen (NDT) [4], [5], followed by secondary metabolism to 4-hydroxy-N-desmethyl-tamoxifen (endoxifen) [6]. 4HT is known to be an effective anti-estrogenic compound since its binding affinity for ERα is approximately 100 fold greater than that of the parent drug as is its ability to suppress estrogen induced cell proliferation rates [7], [8], [9], [10]. For these reasons, the majority of *in vitro* and *in vivo* studies aimed at elucidating the mechanisms of tamoxifen action have focused solely on 4HT. While 4HT continues to be the metabolite commonly employed in preclinical studies, recent reports have confirmed that 4HT plasma concentrations in tamoxifen treated women are very low [11]. In fact, the average steady state circulating levels of tamoxifen, 4HT, and NDT in women receiving the standard dose of tamoxifen therapy (20 mg/day) are 300 nM, 7 nM, and 700 nM respectively [11]. Interestingly, plasma endoxifen concentrations are highly variable, ranging from 5–180 nM, and are associated with cytochrome P450 2D6 (CYP2D6) mediated oxidation of NDT [6].

The identification of endoxifen was originally described in human breast tumor tissue in 1986 [12], however, its pharmacological activity had not been investigated until recently. Endoxifen’s ERα binding affinity, anti-proliferative activity and inhibitory effects on select ERα target genes have been shown to be similar to that of 4HT when administered at equal concentrations [6], [13], [14]. A previous report also suggested that 4HT and endoxifen function similarly in breast cancer cells [15]. However, recent data have demonstrated that the mechanism of action of these two SERMs may differ substantially given that, unlike 4HT or the parent drug tamoxifen, endoxifen uniquely targets ERα for proteasomal degradation similar to that of the ER-down regulator and pure anti-estrogen, ICI 182 780 (ICI) [16]. Additionally, only clinically relevant concentrations of endoxifen, but not 4HT, are able to block estrogen induced changes in gene expression and breast cancer cell proliferation [16]. Furthermore, endoxifen’s anti-estrogenic properties have been shown to be maintained even in the presence of tamoxifen and its other primary metabolites [16]. Based in part on these recent studies, phase I clinical trials of endoxifen are under way at the Mayo Clinic (NCT ID: NCT01327781) and National Cancer Institute (NCT ID: NCT01273168).

Identification of differences in the mechanisms of action of specific anti-estrogens is of importance since estrogen is known to regulate a wide variety of cellular pathways. Elucidation of specific genes and their associated biological pathways that are uniquely regulated by various anti-estrogenic compounds will further enhance our understanding of the way in which these drugs function and could potentially allow us to identify biomarkers that predict a patient’s responsiveness to these compounds. Here, we have compared the ability of 4HT, endoxifen and ICI to target ERα for DNA binding to well characterized target genes and have utilized Illumina HumanHT-12 expression BeadChip arrays to compare and contrast the gene expression profiles of MCF7 cells exposed to equal concentrations of these compounds in the presence and absence of estrogen. Finally, we have determined the concentration dependent effects of endoxifen on global gene expression changes and alterations in the cell cycle under conditions that mimic clinically relevant levels of tamoxifen and its other primary metabolites.

## Results

**Figure 1:**
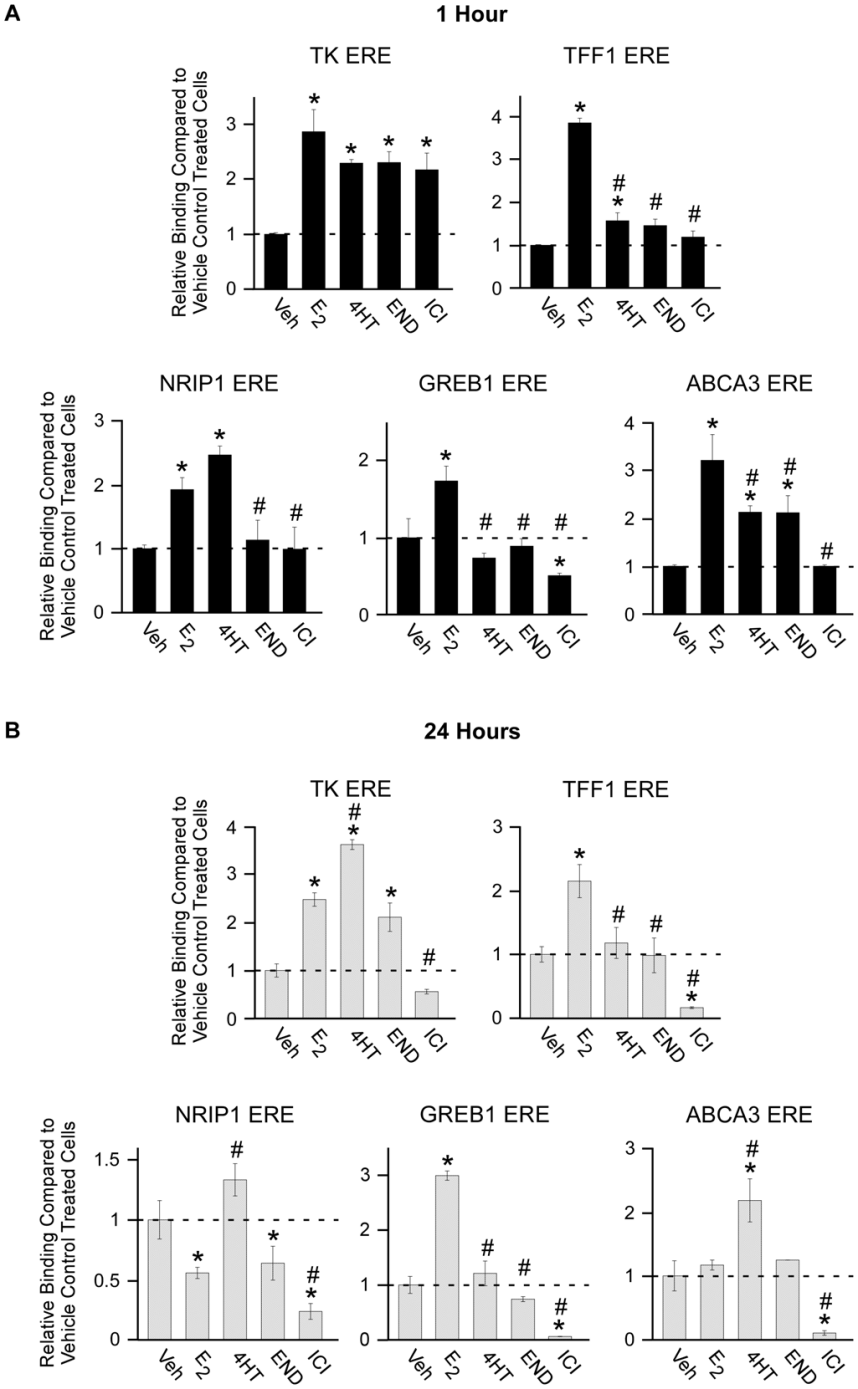
ChIP analysis of ERα binding to a consensus ERE and endogenous target genes. ChIP assays were performed in MCF7 cells transiently transfected with a consensus ERE and treated as indicated for either 1 hour (**A**) or 24 hours (**B**). Data are expressed as the relative abundance of the target following indicated treatments relative to vehicle treated controls as detected by real-time PCR. All data were normalized using input values. Experiments were conducted in triplicate and a representative data set is shown. Asterisks denote significance at the P<0.05 level (ANOVA) compared to vehicle controls. # denotes significant differences (P<0.05) between estrogen and anti-estrogen treatments.

As a first step towards comparing the effects of these compounds in breast cancer cells, we performed ChIP assays to analyze their effects on the recruitment of ERα to a consensus ERE and promoter/enhancer regions in well-characterized endogenous target genes. Following 1 hour of exposure, all ligands led to increased ERα binding to a transiently transfected consensus ERE construct with estrogen treatment resulting in the highest levels of association (Figure 1A). With regard to endogenous target genes, estrogen was shown to enhance ERα association with the known promoter/enhancer regions in the TFF1, NRIP1, GREB1 and ABCA3 genes following 1 hour of treatment (Figure 1A). Treatment with 4HT resulted in enhanced ERα binding to the TFF1, NRIP1 and ABCA3 genes (Figure 1A). Endoxifen treatment only enhanced ERα binding to the ABCA3 gene while ICI treatment did not induce ERα binding to any of these enhancer elements (Figure 1A). After 24 hours of exposure, 4HT treatment resulted in the highest degree of ERα binding to a consensus ERE followed by estrogen and endoxifen (Figure 1B). ICI treatment resulted in further dissociation of ERα binding relative to vehicle treated controls (Figure 1B). With regard to endogenous target genes, ERα binding was maintained on the TFF1 and GREB1 enhancer elements following estrogen treatment, but not on the NRIP1 and ABCA3 genes. Twenty four hours of 4HT treatment induced ERα association with the ABCA3 enhancer region, but not with any of the other target genes (Figure 1B). Endoxifen treatment suppressed ERα binding to the NRIP1 gene and had no effect on the TFF1, GREB1 and ABCA3 enhancer regions relative to vehicle controls (Figure 1B). ICI treatment resulted in dissociation of ERα binding on all 4 enhancer elements (Figure 1B). These data suggest that there are potentially significant differences in the mechanisms by which these ligands function to alter ERα target gene expression. In general, 4HT seemed to enhance ERα association with known EREs in endogenous target genes while endoxifen did not. Similar to endoxifen, ICI treatment had little to no effect on ERα binding following 1 hour of treatment but resulted in significant decreases in ERα association following 24 hours of exposure. Due to the observed differences in the ERα-DNA recruitment profiles elicited by these anti-estrogens, we speculated that the effects of these compounds on the global gene expression profiles of breast cancer cells may also differ substantially.

**Figure 2:**
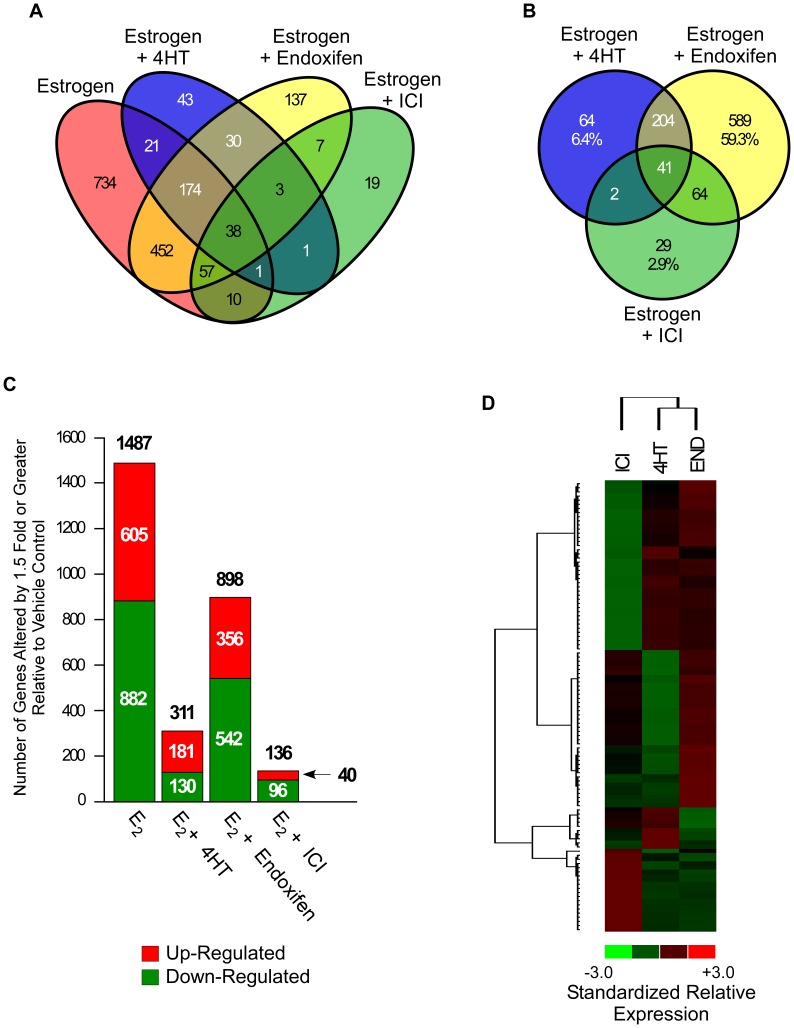
Microarray analysis of 4HT, endoxifen and ICI action in the presence of estrogen. (**A**) Venn diagram of genes whose expression levels were significantly altered by 1.5 fold or greater in MCF7 cells treated with 10 nM estrogen, or estrogen plus 100 nM concentrations of indicated anti-estrogens, relative to vehicle control treated cells following 24 hours of exposure. (**B**) Venn diagram of genes whose expression was altered by estrogen plus anti-estrogen treatments relative to vehicle treatment alone. (**C**) Graph depicting the number of genes up-regulated (red) and down-regulated (green) by indicated treatments relative to vehicle control treated cells. (**D**) Heat map depicting the hierarchical clustering of genes that were differentially expressed in at least one of the indicated treatment groups relative to vehicle control and which had average fold-changes >3 standard deviations from all other genes in the comparison. Red indicates increased gene expression while green indicates decreased gene expression relative to vehicle treated controls.

In order to address this possibility, we compared the gene expression profiles of MCF7 cells exposed to either estrogen (10 nM) alone, or in combination with 100 nM concentrations of 4HT, endoxifen or ICI relative to vehicle controls. As can be seen in Figure 2A, estrogen significantly regulated the expression of 1487 genes, of which 734 (49%) were not altered by the addition of any anti-estrogen. We also identified subsets of genes that were regulated by the addition of 4HT (311 genes), endoxifen (898 genes) or ICI (136 genes) relative to vehicle treated control cells (Figure 2A). Interestingly, the expression profiles of cells treated with endoxifen+estrogen had significantly more overlap with that of cells treated with estrogen alone (724 genes) than with those treated with 4HT (234 genes) or ICI (106 genes) (Figure 2A). A similar Venn diagram was also generated in which the estrogen treatment alone was excluded in order to better visualize differences and similarities between the global gene expression profiles elicited by the three anti-estrogens (Figure 2B). As can be seen, considerable differences were observed between the patterns of gene expression elicited by these compounds in the presence of estrogen with more overlap between the 4HT and endoxifen treatments relative to that of ICI (Figure 2B). Of the genes differentially regulated by an anti-estrogen+estrogen treatment, 64 (6.4%), 589 (59.3%) and 29 (2.9%) were specific to 4HT, endoxifen and ICI respectively (Figure 2B). A list of these genes is provided in Table S1. The total number of genes which were either up-regulated (red) or down-regulated (green) by each treatment is depicted in Figure 2C. Compared to vehicle treatment, estrogen suppressed the expression of 882 genes (59.3%) and induced the expression of 605 genes (40.7%) (Figure 2C). The combination of 4HT and estrogen induced the expression of 181 genes (58.2%) while suppressing 130 (41.8%) (Figure 2C). Endoxifen and ICI combined with estrogen suppressed more genes than they induced. Specifically, endoxifen induced 356 genes (39.6%) and suppressed 542 genes (60.4%) while ICI induced the expression of 40 genes (29.4%) and suppressed 96 (70.6%) compared to vehicle treatment (Figure 2C).

In order to clarify whether the effects of these compounds simply resulted in different degrees of anti-estrogen activity, or whether there were also groups of genes which were uniquely regulated by these ligands, we generated heat maps through hierarchical clustering of this data set. Heat maps were generated using only those genes whose average fold changes across the three treatment replicates were >3 standard deviations from all genes in the comparison in at least one of the treatment groups. This analysis revealed that endoxifen and 4HT treatments cluster more closely together than they do with ICI (Figure 2D). However, it is apparent that there are groups of genes which are uniquely up- or down-regulated by each of these anti-estrogens. Interestingly, there were essentially no genes which were commonly induced or repressed by all three anti-estrogens further suggesting that their mechanisms of action are substantially different (Figure 2D). A heat map showing the clustering and relative expression levels for each of these genes across individual replicate treatments is depicted in Figure S1.

**Figure 3:**
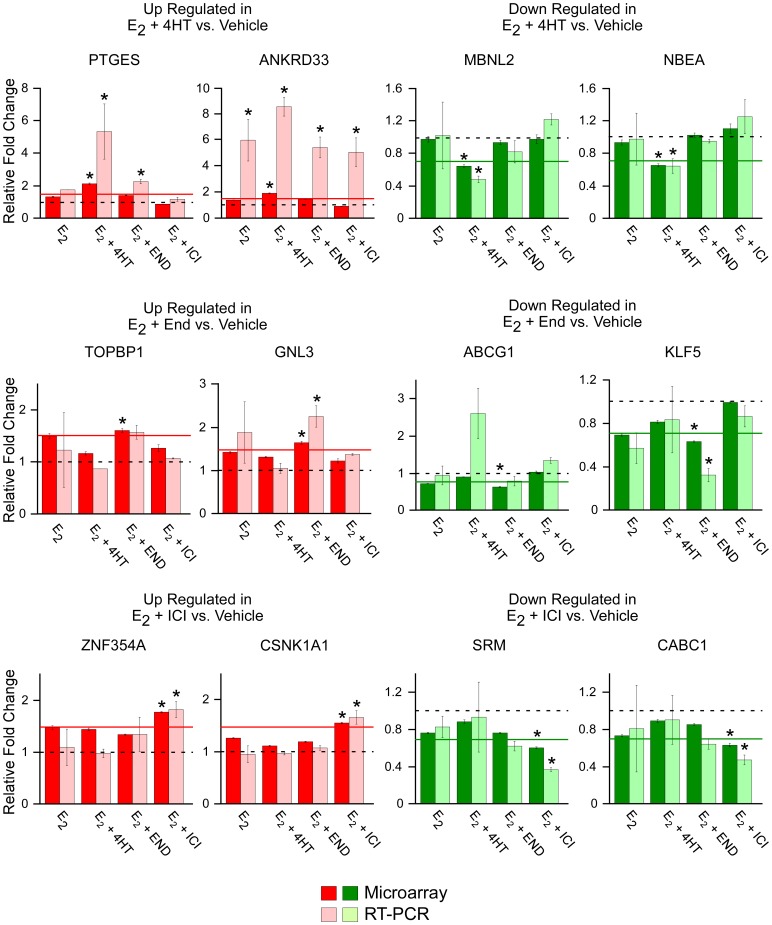
Real-time PCR confirmation of selected genes whose expression levels were either increased (red) or decreased (green) by a specific anti-estrogen. Genes whose expression levels were determined to be specifically increased (red) or decreased (green) by only one of the three anti-estrogen treatments were randomly selected for confirmation of the microarray data. Darkly shaded bars depict relative fold changes from vehicle treated cells (dashed line) as detected by microarray analysis while lightly shaded bars depict fold change as detected by RT-PCR analysis. Solid lines represent the 1.5 fold cut-off used in the microarray analysis. Data represent the mean ± the standard error of three independent treatments. Asterisks denote values with significant differences at the P<0.05 level (ANOVA) relative to vehicle treated controls which also met the 1.5 fold cut-off parameter used in the microarray analysis.

In order to analyze the accuracy of this microarray data and to confirm the observation that subsets of genes which are uniquely and specifically regulated by individual anti-estrogens exist, quantitative RT-PCR was performed on two up-regulated and two down-regulated genes. The results of these studies are depicted in Figure 3 (lightly shaded bars) following normalization to vehicle control treated cells (represented by the dashed line) and are compared to the results obtained via microarray analysis (darkly shaded bars). All 12 of the genes analyzed exhibited similar expression patterns across treatment groups as was detected by microarray analysis confirming the accuracy of our data set (Figure 3). Ten of the 12 genes exhibited significant differences in their treatment group of interest relative to the 1.5 fold cut-off level which was employed in the microarray analysis (represented by the solid line). The two genes which were not significantly regulated compared to the 1.5 fold cut-off (TOPBP1 and ABCG1) were still shown to be specifically regulated by the anti-estrogen of interest (Figure 3). These data support the observation that there are groups of genes whose expression levels are uniquely altered by only one of the three anti-estrogens studied here.

**Figure 4:**
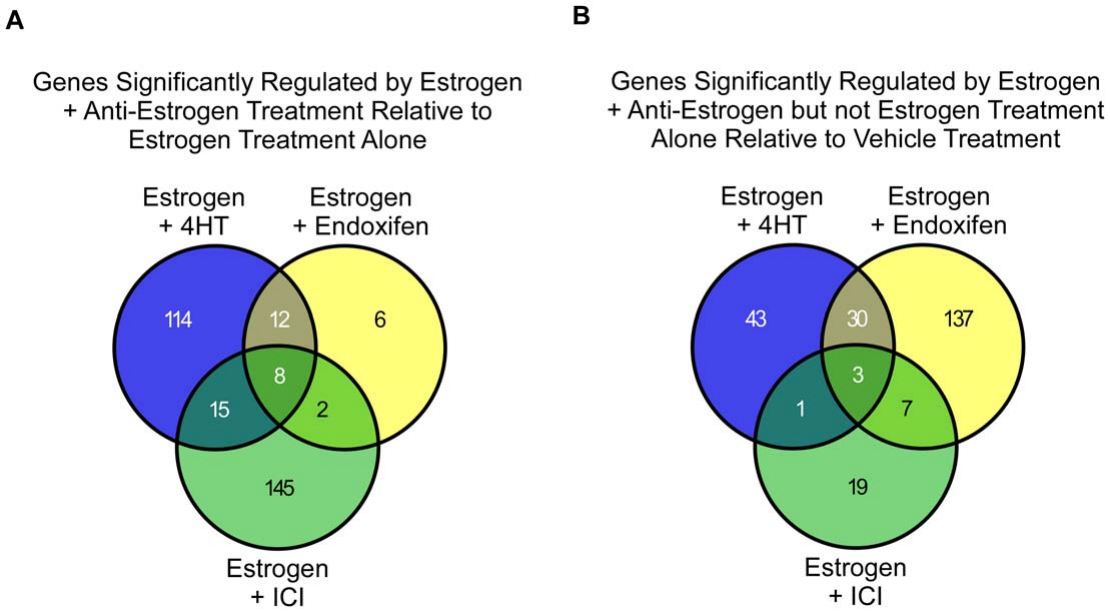
Venn diagrams depicting the anti-estrogen specific effects on estrogen-dependent and -independent genes. (**A**) Venn of genes whose expression levels were significantly altered by 1.5 fold or greater in MCF7 cells treated with 10 nM estrogen plus 100 nM concentrations of indicated anti-estrogens, relative to cells treated with estrogen alone following 24 hours of exposure. (**B**) Venn diagram of genes whose expression levels were significantly altered by 1.5 fold or greater in MCF7 cells treated with 10 nM estrogen plus 100 nM concentrations of indicated anti-estrogens, but not by estrogen treatment alone, relative to vehicle treated controls following 24 hours of exposure.

To further compare the effects of these anti-estrogens with regard to their ability to alter estrogen-mediated gene expression, we developed a Venn diagram using only those genes whose expression levels were significantly altered by 1.5 fold or greater following the addition of an anti-estrogen+estrogen relative to estrogen treatment alone. As shown in Figure 4A, ICI altered the largest number of estrogen-regulated genes (170) followed by 4HT (149). Endoxifen only significantly altered the estrogen-mediated expression levels of 28 genes. A list of these genes is provided in Table S2. Also of note is the fact that there was very little overlap between these gene lists further implying differences in their mechanisms of action.

In light of this observation, and the data presented in Figure 2, we next generated a Venn diagram using only those genes that were determined to be significantly regulated by estrogen +4HT, estrogen+endoxifen or estrogen+ICI, but not by estrogen treatment alone. This analysis revealed that the addition of ICI had very little influence on the expression of non-estrogen regulated genes (30 genes total) followed by 4HT (77 genes) (Figure 4B). Endoxifen was shown to have a much more substantial effect as it regulated 177 genes which were not regulated by estrogen treatment alone (Figure 4B). A list of these genes is provided in Table S3. These data suggest that these anti-estrogens, particularly endoxifen, likely have functions which are independent of simply reversing estrogen-mediated effects at the level of gene expression.

**Figure 5:**
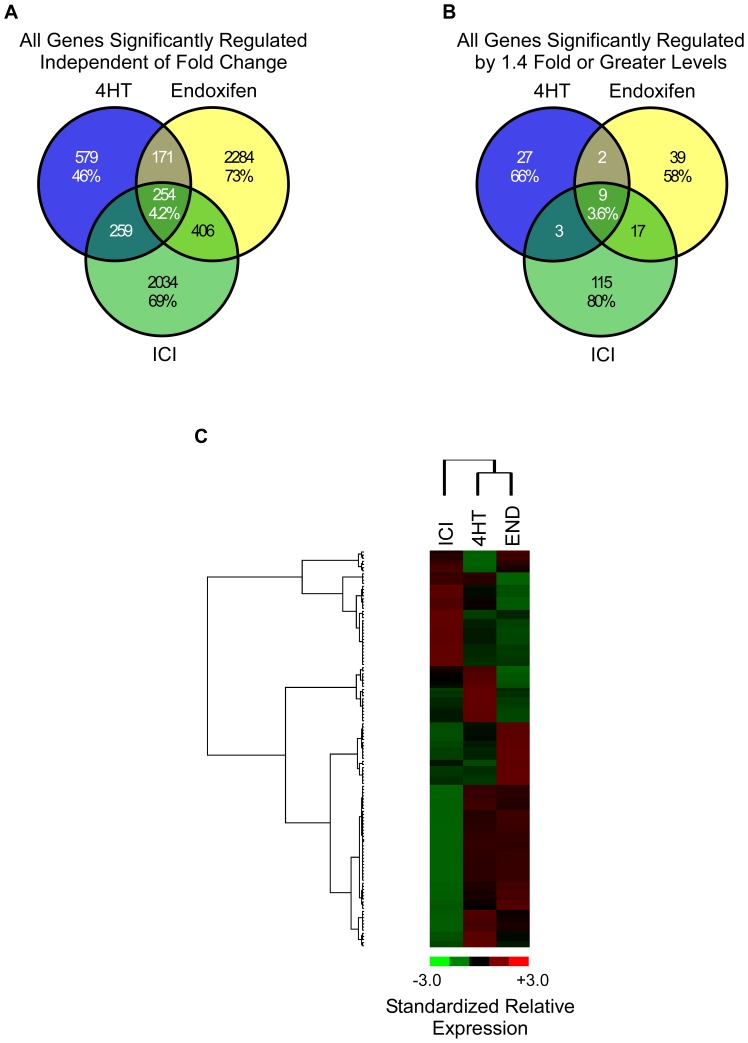
Microarray analysis of 4HT, endoxifen and ICI action in the absence of estrogen. (**A**) Venn diagram of genes whose expression levels were determined to be significantly altered by 100 nM concentrations of 4HT, endoxifen or ICI in the absence of estrogen following 24 hours of regardless of fold-change levels. (**B**) A second Venn diagram was developed using only those genes which exhibited fold-changes of 1.4 fold or greater relative to vehicle treated control cells. (**C**) Heat map depicting the hierarchical clustering of genes that were differentially expressed in at least one of the indicated treatment groups relative to vehicle control and which had average fold-changes >3 standard deviations from all other genes in the comparison. Red indicates increased gene expression while green indicates decreased gene expression relative to vehicle treated controls.

In order to address this possibility, we next examined the global gene expression changes of MCF7 cells following 24 hour treatments with 100 nM concentrations of 4HT, endoxifen or ICI in the absence of estrogen. A Venn diagram was first created using all genes determined to be significantly regulated by one or more of these anti-estrogens regardless of fold-change relative to vehicle treated controls. As shown in Figure 5A, 4HT, endoxifen and ICI were shown to regulate a substantial number of genes, many of which were unique to a given treatment. Specifically, 579 (46%), 2284 (73%) and 2034 (69%) genes were uniquely regulated by 4HT, endoxifen or ICI respectively with only 254 (4.2%) genes detected to be altered by all three compounds (Figure 5A). Another Venn diagram was created using only those genes that were determined to be significantly regulated by at least one treatment and which exhibited fold-changes of 1.4 or greater relative to vehicle treated cells. A fold-change of 1.4 instead of 1.5 was chosen as a cut-off in order to include a larger number of genes in the comparison. This analysis resulted in a similar profile of gene expression changes with 66%, 58% and 80% being unique to 4HT, endoxifen and ICI treatment respectively with very little overlap between the three compounds (Figure 5B). A list of these genes is provided in Table S4. Similar to the data presented in Figure 2D, heat map analysis using averaged fold changes for differentially regulated genes demonstrated that endoxifen and 4HT again clustered more closely with one another than they did with ICI (Figure 5C). However, it should be noted that clusters of genes which were specifically up- or down-regulated by each treatment were identified (Figure 5C). A heat map showing the clustering and relative expression levels for each of these genes across individual replicate treatments is depicted in Figure S2.

**Figure 6:**
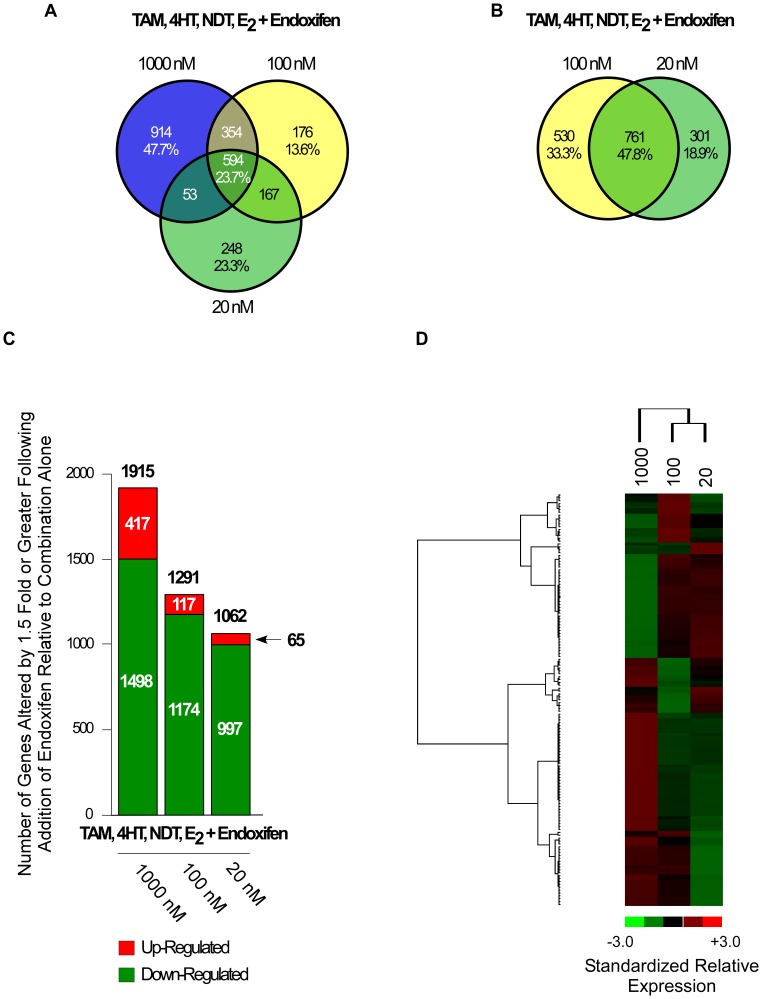
Microarray analysis of endoxifen concentration-dependent changes in gene expression in the presence of estrogen and physiologically relevant levels tamoxifen and its metabolites. (**A**) Venn diagram of genes whose expression levels were significantly altered by 1.5 fold or greater in MCF7 cells treated with 10 nM estrogen (E_2_), 300 nM tamoxifen (TAM), 7 nM 4-hydroxy-tamoxifen (4HT) and 700 nM N-desmethyl-tamoxifen (NDT), plus indicated concentrations of endoxifen, relative to cells treated with all compounds minus endoxifen for 24 hours. (**B**) Venn diagram of genes detected to be regulated by the 100 nM and 20 nM endoxifen treatments. (**C**) Graph depicting the number of genes up-regulated (red) and down-regulated (green) by indicated treatments relative to E_2_, TAM, 4HT and NDT treated cells. (**D**) Heat map depicting the hierarchical clustering of genes that were differentially expressed in at least one of the indicated treatment groups relative to E_2_, TAM, 4HT and NDT treated cells and which had average fold-changes >3 standard deviations from all other genes in the comparison. Red indicates increased gene expression while green indicates decreased gene expression relative to vehicle treated controls.

As a final global analysis of endoxifen action in breast cancer cells, we sought to determine if changes in endoxifen concentration would significantly impact the gene expression profiles under conditions that mimic the steady-state concentrations of tamoxifen and its metabolites that are observed in women receiving a 20 mg/day dose of tamoxifen [11]. MCF7 cells were treated with estrogen (10 nM), tamoxifen (300 nM), NDT (700 nM) and 4HT (7 nM), and one of three endoxifen concentrations (20, 100 or 1000 nM). These endoxifen concentrations correspond to a CYP2D6 poor metabolizer, a high intermediate to extensive metabolizer, and to a pharmacological dose of endoxifen respectively. Following 24 hour treatments, microarray analyses were conducted and gene expression changes elicited by the addition of the various endoxifen concentrations were determined relative to cells treated with estrogen, tamoxifen, NDT and 4HT alone. Interestingly, of all the genes detected to be differentially regulated, 248 (23.3%), 176 (13.6%), and 914 (47.7%) were specific to the addition of 20, 100 or 1000 nM endoxifen respectively while only 594 (23.7%) genes were commonly regulated by all three endoxifen concentrations (Figure 6A). A list of these genes is provided in Table S5. Comparison of the two endoxifen concentrations mimicking an extensive and poor metabolizer revealed an overlap of 761 (47.8%) genes, however, 530 (33.3%) genes and 301 (18.9%) genes were unique to the 100 nM and 20 nM endoxifen concentrations respectively (Figure 6B). A list of these genes is also provided in Table S5.

The total number of genes which were either up-regulated (red) or down-regulated (green) by each treatment is depicted in Figure 6C. When compared to cells treated with estrogen, tamoxifen, NDT and 4HT, the addition of any concentration of endoxifen led to substantially more gene suppression than gene induction. More specifically, 1498 (78.2%), 1174 (90.9%) and 997 (93.9%) genes were suppressed by the addition of 1000 nM, 100 nM and 20 nM endoxifen respectively (Figure 6C). Interestingly, higher concentrations of endoxifen resulted in the induction of more genes compared to lower concentrations as 417 (21.8%), 117 (9.1%) and 65 (6.1%) genes were up-regulated by 1000 nM, 100 nM and 20 nM endoxifen respectively (Figure 6C). Heat map analysis using averaged fold changes for differentially regulated genes revealed clusters of genes which are uniquely up- or down-regulated by a specific endoxifen concentration (Figure 6D). A heat map showing the clustering and relative expression levels for each of these genes across individual replicate treatments is depicted in Figure S3.

**Figure 7:**
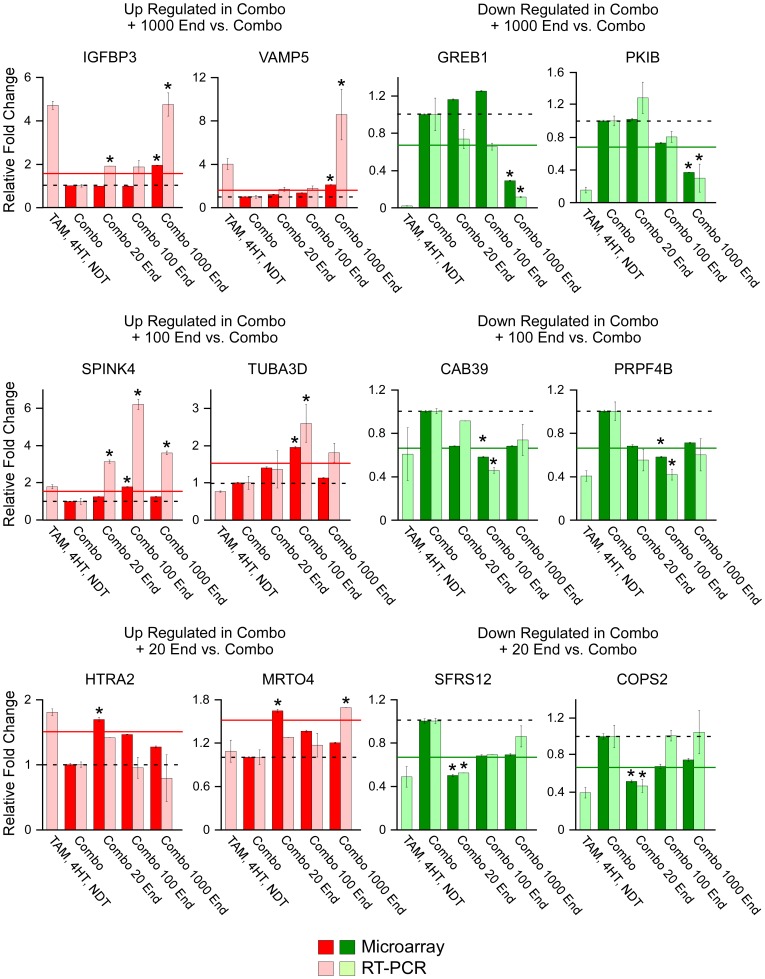
Real-time PCR confirmation of randomly selected genes whose expression levels were either increased (red) or decreased (green) by a specific endoxifen concentration. Genes whose expression levels were determined to be specifically increased (red) or decreased (green) by only one of the three endoxifen concentrations were randomly selected for confirmation of the microarray data. Darkly shaded bars depict relative fold changes from E_2_ (10 nM), TAM (300 nM), 4HT (7 nM) and NDT (700 nM) treated cells (combo/dashed line) as detected by microarray analysis while lightly shaded bars depict fold change as detected by RT-PCR analysis. Solid lines represent the 1.5 fold cut-off used in the microarray analysis. Data represent the mean ± the standard error of three independent treatments. Asterisks denote values with significant differences at the P<0.05 level (ANOVA) relative to combo treated controls which also met the 1.5 fold cut-off parameter used in the microarray analysis.

As above, quantitative RT-PCR was performed on two of these uniquely up-regulated or down-regulated genes from each of the three endoxifen concentrations. The results of these studies are depicted in Figure 7 (lightly shaded bars) following normalization to cells treated with TAM, 4HT, NDT and estrogen (represented by the dashed line) and are compared to the results obtained via microarray analysis (darkly shaded bars). Although microarray analysis was not performed on cells treated with only TAM, 4HT and NDT, we have included RT-PCR data for this treatment group to allow for determination of estrogen-specific effects. All 12 of the genes analyzed exhibited similar expression patterns across treatment groups as was detected by microarray analysis confirming the accuracy of our data set (Figure 7). Of the 12 genes analyzed, only one (MRTO4) did not match the endoxifen concentration specific profile as detected by microarray analysis. A second gene, HTRA2, was not significantly different from the microarray cut-off of 1.5 fold (represented by the solid line); however, it was still shown to be most highly regulated by the 20 nM concentration of endoxifen as was detected in the microarray analysis. These data support the notion that alterations in endoxifen concentration, even in the presence of tamoxifen and its other metabolites, have significant and unique impacts on the global gene expression profiles of breast cancer cells.

**Figure 8:**
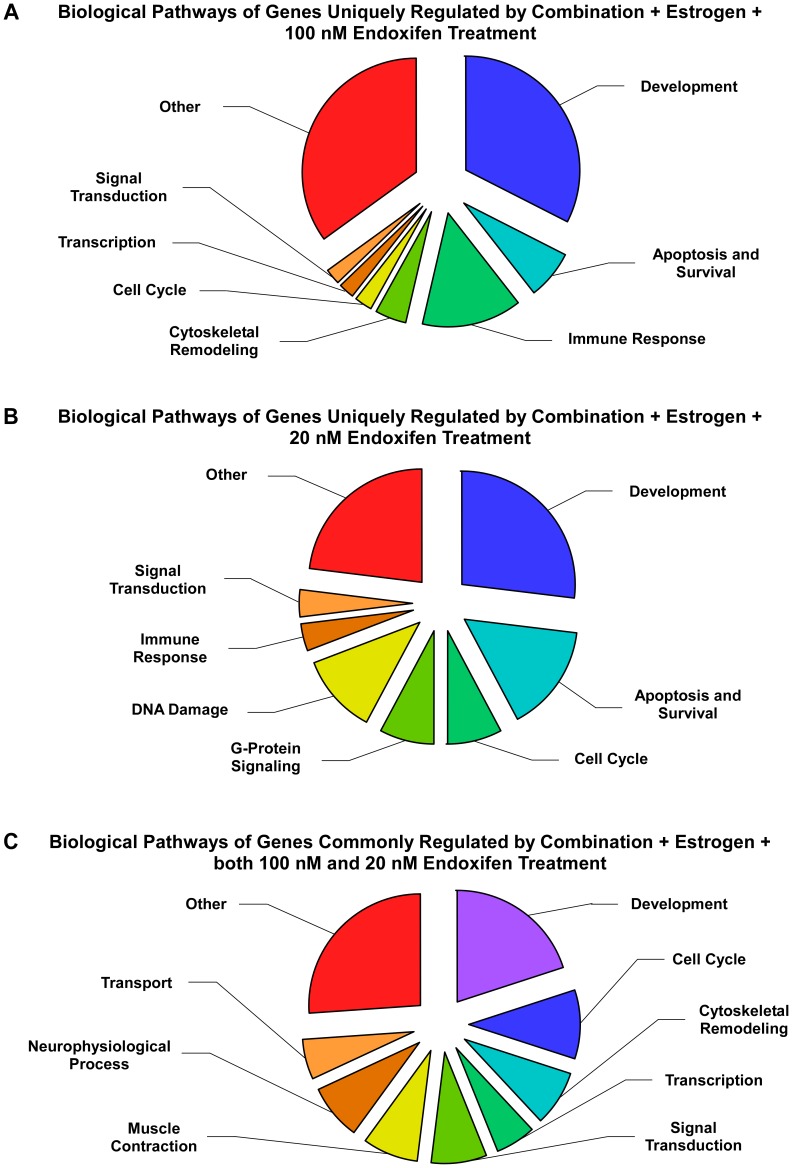
Pathway analysis of genes regulated by 100 nM or 20 nM endoxifen treatments in the presence of estrogen and physiologically relevant levels tamoxifen and its metabolites. The sub-categories of biological pathways determined to be significantly altered and which were unique to the 100 nM endoxifen treatment (**A**) or the 20 nM endoxifen treatment (**B**), or which were commonly regulated by both endoxifen concentrations (**C**), are shown. The specific biological pathways which are comprised within these sub-categories are listed in Table 1.

We next performed pathway analysis on the genes that were uniquely and commonly regulated by the treatments mimicking the extensive (100 nM) and poor (20 nM) metabolizers in order to identify specific biological functions that were affected by these two different endoxifen concentrations. This analysis revealed that not only do the gene expression profiles differ based on endoxifen concentrations, but the biological pathways of MCF7 cells are also differentially affected. Figure 8 depicts the major types of biological pathways that were altered by each treatment with the size of each portion of the pie graphs corresponding to the number of sub-categories comprising each pathway. While these global categories appear to be somewhat similar, the specific sub-categories affected by these endoxifen concentrations are very different (Table 1). Specifically, 37 out of the 43 pathways altered by 100 nM endoxifen were unique to this treatment and are indicated by an asterisk in Table 1. Included in this list were molecular processes relating to apoptosis, survival and proliferation as well as a number of signaling pathways such as Flt3, Igf1, EGFR, GM-CSF, MapK, IL-4, P53, GDNF and VEGF (Table 1). With regard to the addition of 20 nM endoxifen, 19 out of the 26 pathways altered were unique to this treatment and are also indicated by an asterisk in Table 1. Included in this list were several DNA damage related processes and a number of cell cycle pathways (Table 1). Pathways commonly regulated by both 100 nM and 20 nM endoxifen also included a number of cell cycle related processes in addition to transcription, translation, transport and migration related pathways among others (Table 1).

**Table 1:** Biological pathways detected to be significantly regulated based upon endoxifen concentrations under physiologically relevant conditions.

Pathway #		Pathway Name	P-Value	# Genes	
Pathways of Genes Uniquely Regulated by Combination+Estrogen+100 nM Endoxifen					
1*		Development_Flt3 signaling	0.0007298	6/41	
2*		Mechanisms of CFTR activation by S-nitrosoglutathione (normal and CF)	0.001463	4/19	
3		Apoptosis and survival_BAD phosphorylation	0.00261	5/36	
4*		Development_A2A receptor signaling	0.00261	5/36	
5*		dATP/dITP metabolism	0.003148	6/54	
6*		Apoptosis and survival_Anti-apoptotic action of membrane-bound ESR1	0.003612	4/24	
7*		Development_SSTR2 in regulation of cell proliferation	0.004211	4/25	
8*		Development_IGF-1 receptor signaling	0.006329	5/44	
9*		Development_EGFR signaling via small GTPases	0.007273	4/29	
10*		Development_GM-CSF signaling	0.008383	5/47	
11*		Translation _Regulation activity of EIF4F	0.009983	5/49	
12		Cell cycle_Spindle assembly and chromosome separation	0.01035	4/32	
13*		Development_PDGF signaling via MAPK cascades	0.0128	4/34	
14		Development_VEGF signaling and activation	0.0128	4/34	
15*		Immune response_IL-4 signaling pathway	0.01416	4/35	
16*		Cytoskeleton remodeling_Role of PDGFs in cell migration	0.01821	3/21	
17		Aspartate and asparagine metabolism	0.01821	3/21	
18*		TTP metabolism	0.01876	4/38	
19*		Development_A1 receptor signaling	0.01876	4/38	
20*		dGTP metabolism	0.01876	4/38	
21*		Transcription_P53 signaling pathway	0.02047	4/39	
22		Signal transduction_AKT signaling	0.02047	4/39	
23*		Aminoacyl-tRNA biosynthesis in cytoplasm/Rodent version	0.02067	3/22	
24*		Oxidative stress_Role of ASK1 under oxidative stress	0.02067	3/22	
25*		Aminoacyl-tRNA biosynthesis in cytoplasm	0.02067	3/22	
26*		Development_GDNF family signaling	0.02228	4/40	
27*		Immune response_CD28 signaling	0.02619	4/42	
28		Development_FGFR signaling pathway	0.02829	4/43	
29*		Immune response_TREM1 signaling pathway	0.03278	4/45	
30*		Cholesterol and Sphingolipids transport/Influx to the early endosome in lung	0.03432	2/11	
31*		Immune response_IL-2 activation and signaling pathway	0.03517	4/46	
32*		dCTP/dUTP metabolism	0.03517	4/46	
33*		Regulation of lipid metabolism_Insulin regulation of fatty acid methabolism	0.03517	4/46	
34*		Immune response_IL-4 - antiapoptotic action	0.03561	3/27	
35*		Some pathways of EMT in cancer cells	0.04025	4/48	
36*		Phospholipid metabolism p.1	0.04047	2/12	
37*		Immune response_PIP3 signaling in B lymphocytes	0.04279	3/29	
38*		Apoptosis and survival_p53-dependent apoptosis	0.04279	3/29	
39*		Development_VEGF signaling via VEGFR2 - generic cascades	0.04279	3/29	
40*		Development_PDGF signaling via STATs and NF-kB	0.04279	3/29	
41*		Cytoskeleton remodeling_Cytoskeleton remodeling	0.04526	6/96	
42*		Development_CNTF receptor signaling	0.04663	3/30	
43*		Keratan sulfate metabolism p.1	0.047	2/13	
**Pathway #**		**Pathway Name**	**P-Value**	**# Genes**	
**Pathways of Genes Uniquely Regulated by Combination+Estrogen+20 nM Endoxifen**					
1*		Immune response_CD40 signaling	0.002114	5/54	
2		Apoptosis and survival_BAD phosphorylation	0.003086	4/36	
3		G-protein signaling_Proinsulin C-peptide signaling	0.003769	4/38	
4		Development_FGFR signaling pathway	0.005909	4/43	
5*		Development_Glucocorticoid receptor signaling	0.006685	3/23	
6*		DNA damage_ATM/ATR regulation of G2/M checkpoint	0.009461	3/26	
7*		DNA damage_DNA-damage-induced responses	0.009651	2/9	
8*		Cell cycle_Role of SCF complex in cell cycle regulation	0.01282	3/29	
9		Development_EGFR signaling via small GTPases	0.01282	3/29	
10*		Apoptosis and survival_Role of IAP-proteins in apoptosis	0.01282	3/29	
11*		DNA damage_Brca1 as a transcription regulator	0.01407	3/30	
12*		G-protein signaling_G-Protein alpha-12 signaling pathway	0.01678	3/32	
13*		Cell cycle_Role of APC in cell cycle regulation	0.01678	3/32	
14*		Histidine-glutamate-glutamine metabolism	0.01823	3/33	
15*		Apoptosis and survival_Ceramides signaling pathway	0.01975	3/34	
16*		Apoptosis and survival_DNA-damage-induced apoptosis	0.02634	2/15	
17		Signal transduction_AKT signaling	0.0284	3/39	
18*		Development_Thrombopoietin-regulated cell processes	0.0344	3/42	
19*		Blood coagulation_GPVI-dependent platelet activation	0.03654	3/43	
20*		Development_TGF-beta-dependent induction of EMT via RhoA, PI3K and ILK.	0.03654	3/43	
21		Transport_RAN regulation pathway	0.03714	2/18	
22*		Development_FGF2-dependent induction of EMT	0.03714	2/18	
23*		Histidine-glutamate-glutamine and proline metabolism/Rodent version	0.04101	3/45	
24*		Development_EGFR signaling via PIP3	0.04106	2/19	
25*		Gamma-aminobutyrate (GABA) biosynthesis and metabolism	0.04513	2/20	
26		Aspartate and asparagine metabolism	0.04934	2/21	
**Pathway #**		**Pathway Name**	**P-Value**	**# Genes**	
**Pathways of Genes Commonly Regulated by Combination+Estrogen+both 100 and 20 nM Endoxifen**					
1	Development_EDG3 signaling pathway		3.144E−06		8/26
2	Muscle contraction_Regulation of eNOS activity in endothelial cells		7.175E−06		10/47
3	Muscle contraction_EDG5-mediated smooth muscle contraction		1.047E−05		7/22
4	Neurophysiological process_ACM regulation of nerve impulse		2.819E−05		8/34
5	Muscle contraction_ACM regulation of smooth muscle contraction		3.538E−05		8/35
6	Cytoskeleton remodeling_ACM3 and ACM4 in keratinocyte migration		9.185E−05		6/21
7	Development_Activation of astroglial cells proliferation by ACM3		9.185E−05		6/21
8	Transport_ACM3 in salivary glands		0.0002642		6/25
9	Cell cycle_Role of Nek in cell cycle regulation		0.0006248		6/29
10	Cell cycle_Chromosome condensation in prometaphase		0.0007215		5/20
11	Signal transduction_Erk Interactions: Inhibition of Erk		0.0007568		6/30
12	Muscle contraction_ GPCRs in the regulation of smooth muscle tone		0.0008466		8/54
13	Cell cycle_Spindle assembly and chromosome separation		0.001085		6/32
14	Transcription_ChREBP regulation pathway		0.001093		4/13
15	Development_G-Proteins mediated regulation MARK-ERK signaling		0.001768		6/35
16	Cytoskeleton remodeling_ESR1 action on cytoskeleton remodeling		0.001965		4/15
17	Apoptosis and survival_Beta-2 adrenergic receptor anti-apoptotic action		0.001965		4/15
18	Cell cycle_The metaphase checkpoint		0.002057		6/36
19	G-protein signaling_Proinsulin C-peptide signaling		0.002738		6/38
20	Development_EDG1 signaling pathway		0.00303		5/27
21	Development_Alpha-2 adrenergic receptor activation of ERK		0.003576		6/40
22	Transport_RAN regulation pathway		0.004031		4/18
23	Regulation of lipid metabolism_Regulation of lipid metabolism		0.004193		5/29
24	Neurophysiological process_NMDA-dependent postsynaptic potentiation		0.004812		7/56
25	Neurophysiological process_Glutamate regulation of Dopamine D1A receptor		0.005644		5/31
26	Cytoskeleton remodeling_Role of PKA in cytoskeleton reorganisation		0.005644		5/31
27	Signal transduction_Activation of PKC via G-Protein coupled receptor		0.005805		6/44
28	Signal transduction_Calcium signaling		0.006488		5/32
29	Cell cycle_Sister chromatid cohesion		0.007215		4/21
30	G-protein signaling_EDG5 signaling		0.007215		4/21
31	Development_Role of HDAC and CaMK in control of skeletal myogenesis		0.008043		6/47
32	Signal transduction_cAMP signaling		0.008435		5/34
33	Immune response_Alternative complement pathway		0.008435		5/34
34	Development_VEGF signaling and activation		0.008435		5/34
35	Regulation of lipid metabolism_Stimulation of Arachidonic acid production		0.008911		6/48
36	Cytoskeleton remodeling_Alpha-1A adrenergic receptor inhibition of PI3K		0.00917		3/12
37	Transport_RAB1A regulation pathway		0.00917		3/12
38	Transcription_Receptor-mediated HIF regulation		0.009546		5/35
39	Development_Endothelin-1/EDNRA transactivation of EGFR		0.009546		5/35
40	Oxidative stress_Angiotensin II-induced production of ROS		0.01006		4/23
41	Apoptosis and survival_BAD phosphorylation		0.01075		5/36
42	Translation _Regulation of translation initiation		0.01173		4/24
43	Cardiac Hypertrophy_Ca(2+)-dependent NF-AT signaling		0.01206		5/37
44	Transcription_CREB pathway		0.01206		5/37
45	Cell adhesion_Histamine H1 receptor signaling		0.01206		5/37
46	Translation_Insulin regulation of translation		0.01348		5/38
47	Neurophysiological process_Dopamine D2 receptor signaling in CNS		0.01356		4/25
48	Development_Angiotensin activation of Akt		0.01356		4/25
49	Blood coagulation_GPCRs in platelet aggregation		0.01428		6/53
50	Development_EDG6 signaling pathway		0.01435		3/14

**Figure 9:**
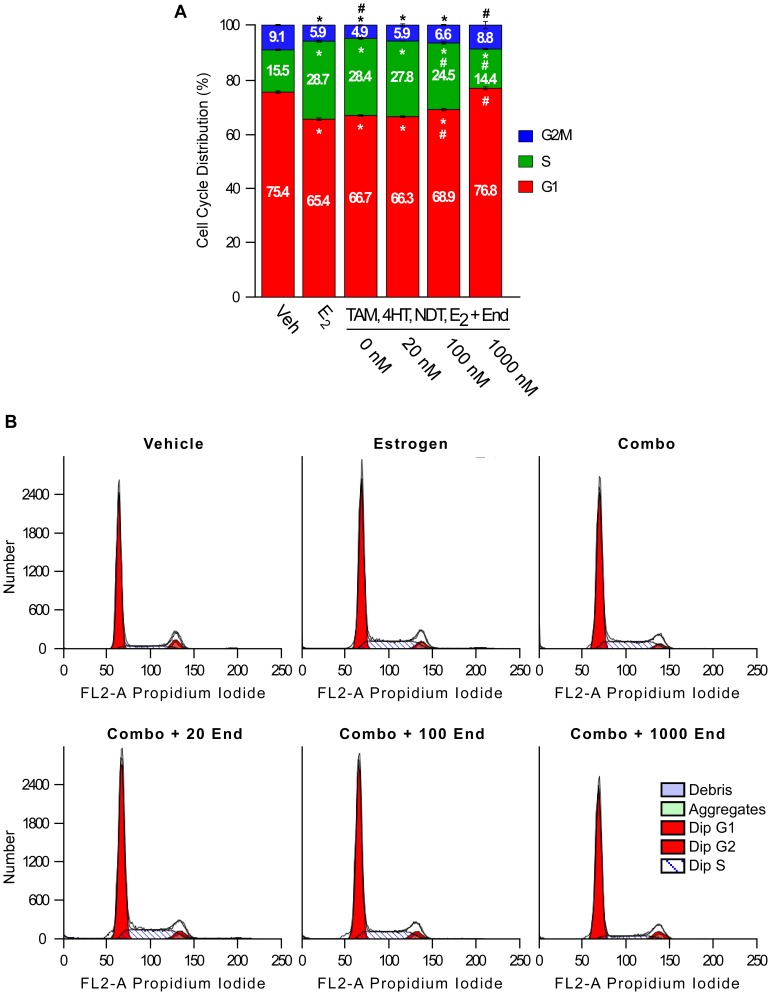
Profile of cell cycle changes induced by endoxifen. MCF7 cells were treated as indicated for 24 hours and cell cycle profiles were determined by propidium iodide staining and flow cytometry. (**A**) The percentage of cells from each treatment in G2/M phase (blue), S phase (green) and G1 phase (red) are shown. Asterisks within each cell cycle phase denote significance at the P<0.05 level (ANOVA) compared to vehicle controls. # within each cell cycle phase denotes significant differences (P<0.05) compared to estrogen treated cells. (**B**) Representative flow cytometry plots for each treatment condition.

Since pathways relating to cell cycle progression and proliferation were among the most abundantly regulated biological processes following endoxifen exposure, we sought to characterize the effects of various endoxifen concentrations on MCF7 cell cycle distribution in the presence of estrogen and clinically relevant concentrations of tamoxifen and its other metabolites. As expected, estrogen treatment alone significantly increased the percentage of cells in S phase and decreased the percentage of cells in G1 and G2/M phase (Figure 9). The addition of tamoxifen (300 nM), NDT (700 nM) and 4HT (7 nM) to the estrogen treatment had little to no effect on the distribution of cells across these cell cycle phases (Figure 9). The addition of 20 nM levels of endoxifen to this combination treatment also had no significant effect (Figure 9). However, progressive and significant decreases in the percentage of cells in S phase were observed following the addition of 100 nM and 1000 nM endoxifen with concomitant increases in G1 and G2/M phases (Figure 9).

## Discussion

The results of our studies strongly suggest that the molecular mechanisms of action between 4HT, endoxifen and ICI in breast cancers cells are markedly different. Through the use of microarray analyses, we have shown that the overlap in gene expression between these three anti-estrogens is relatively low with the large majority of genes being specifically regulated by individual compounds. We have also demonstrated that the gene expression patterns of MCF7 cells vary widely as a function of endoxifen concentration even in the presence of clinically relevant levels of tamoxifen and its other primary metabolites. These differential effects are further exemplified by the fact that the large majority of biological pathways identified through gene clustering are endoxifen concentration dependent. Finally, alterations in cell cycle progression were shown to be dependent upon endoxifen concentration with little to no influence from tamoxifen, NDT and 4HT. Taken together, these data support those of Madlensky et al demonstrating the importance of endoxifen concentrations in tamoxifen treated women [17] and suggest that endoxifen may result in antitumor activity in patients refractory to tamoxifen, fulvestrant and other anti-estrogenic compounds due to its differential mechanisms of action, a concept that is currently being tested in clinical trials.

SERMs, including tamoxifen, 4HT, and NDT, and pure anti-estrogens such as ICI, are known to function by blocking estrogen binding to ERα [18] or by targeting it for proteasomal degradation [19]. Previous studies have demonstrated that ERα associates with numerous, but variable, DNA binding sites in MCF7 cells following tamoxifen [20], [21] and ICI [21] treatment. We therefore compared the ability of endoxifen to target ERα for binding to well characterized promoter/enhancer regions of target genes with that of 4HT and ICI. Our data reveal that endoxifen induces DNA binding of ERα to a consensus ERE at levels similar to that of 4HT and ICI following 1 hour of treatment and intermediate to that of 4HT and ICI following 24 hours of treatment. With respect to endogenous target genes, 4HT treatment was shown to generally enhance ERα binding at both time points while ICI either had no effect or suppressed ERα binding. Interestingly, endoxifen had little to no effect on ERα association with these enhancer elements when compared to vehicle treated control cells suggesting that it may target ERα for unique sites across the genome. This differential binding by the receptor in response to these compounds could partially be explained by the fact that 4HT stabilizes ERα protein levels in MCF7 cells while ICI rapidly targets it for degradation with intermediate effects elicited by endoxifen [16]. Additionally, it is likely that these compounds also have differential effects on the recruitment of both co-activators and co-repressors to these target sites, a possibility that would be of interest to examine in the future. The fact that ERα/DNA interactions following endoxifen treatment are different than that of 4HT and ICI suggested that the patterns of gene expression elicited by these anti-estrogens may also vary.

Through the use of microarray analyses, we have demonstrated that treatment of MCF7 cells with equimolar concentrations of 4HT, endoxifen and ICI in the presence of estrogen for 24 hours resulted in significant differences in gene expression. In fact, of the 993 total genes regulated, only 41 (4.1%) were altered by all three compounds. Furthermore, the overlap between endoxifen regulated genes and 4HT or ICI was 25.4% and 11.3% respectively. Specifically, 898 genes were regulated following estrogen plus endoxifen while only 311 and 136 genes were regulated by the addition of 4HT or ICI relative to vehicle treated controls. Hierarchical clustering of these data revealed that the gene expression profiles elicited by endoxifen and 4HT have more similarities to each other than with that of ICI. However, clusters of genes which were only induced or repressed by one of the three treatments were identified. Of particular interest was the observation that there were no gene clusters which were commonly regulated (in the same direction) by all three anti-estrogens lending further support to the notion that these drugs utilize different mechanisms of action.

A closer examination of these gene expression data revealed that ICI is the most potent anti-estrogen as it altered the largest number of estrogen regulated genes followed by 4HT and endoxifen. Surprisingly, and in spite of the facts that endoxifen’s ERα binding affinity, anti-proliferative activity and inhibitory effects on select ERα target genes are similar to that of 4HT and ICI when administered at equal concentrations [6], [13], [14], endoxifen was shown to alter the fewest number of estrogen-regulated genes in the analyses conducted here. Taken together, these results suggest that endoxifen may have functions which are independent of the estrogen receptor. This possibility is supported by the observation that endoxifen+estrogen treatment resulted in the regulation of substantially more genes that were not regulated by estrogen treatment alone relative to that of 4HT and ICI. The fact that there were a large number of genes (1185) that were significantly regulated by estrogen treatment alone, but were not significantly affected by the addition of any anti-estrogen, may be explained by our use of 10 nM concentrations of estrogen and 100 nM concentrations of each anti-estrogen. Microarray analysis of MCF7 cells treated only with 4HT, endoxifen or ICI in the absence of estrogen revealed that each of these compounds significantly regulated a substantial number of genes, the majority of which were specific to a given treatment. These data further suggest that their mechanisms of action in breast cancer cells are unique and that they may have additional functions aside from solely modulating estrogen-mediated effects.

Our findings demonstrating a substantial difference between 4HT and endoxifen mediated gene expression changes contrast somewhat with the data generated by Lim et al. [15] whose report suggested that 4HT and endoxifen regulate similar patterns of genes. However, it is important to note that there are a number of experimental factors that could potentially explain these differences. First, we have utilized pure Z-isomers of endoxifen as this is the form generated in tamoxifen-treated humans. In the report by Lim et al., a mixture of 75% Z-form and 25% E-form endoxifen was used. Second, the concentrations of estrogen used were different. Lim et al., utilized 0.1 nM concentrations of 17-β-estradiol while we used 10 nM concentrations. The higher concentrations used here were selected in order to maximize the estrogenic response. Since the relative binding affinities of estrogen, 4HT, endoxifen and ICI for ERα are similar; we have effectively increased the competition for ERα binding between estrogen and these anti-estrogens in our study. Third, Lim et al., employed the Affymetrix U133A GeneChips that contain 14,500 genes, while we used the Illumina HumanHT-12 expression BeadChip platform that targets more than 25,000 annotated genes.

In an additional set of studies, we sought to determine if alterations in endoxifen concentrations could significantly impact gene expression profiles, even in the presence of estrogen, tamoxifen and the other tamoxifen metabolites. Our treatment conditions were aimed at mimicking the clinically relevant endoxifen concentrations observed in CYP2D6 poor and extensive metabolizers, as well as a pharmacologic dose of endoxifen. Our results clearly demonstrate that changes in endoxifen levels dramatically impact the gene expression profiles, biological pathways and cell cycle progression of MCF7 cells with the highest concentration (1000 nM) having the greatest impact. Additionally, there were numerous differences between the two endoxifen concentrations aimed at recapitulating a poor (20 nM) vs. extensive (100 nM) metabolizer. These data provide further evidence that endoxifen concentrations are likely to substantially contribute to the overall effectiveness of tamoxifen in breast cancer patients.

In summary, our data clearly demonstrate that the molecular mechanisms of endoxifen action differ from those of 4HT and ICI, and provide compelling evidence that the relative concentrations of endoxifen are likely to affect the activity and side effects of tamoxifen therapy as outlined by Madlensky and colleagues [17]. Differences in endoxifen concentrations in tamoxifen-treated patients could also significantly impact the type of resistance that eventually develops. These data continue to support the ongoing clinical trials of endoxifen which seek to identify its utility for the treatment of endocrine responsive, and potentially “endocrine resistant”, breast cancers. Finally, our studies suggest that higher doses of endoxifen (1 µM or greater), which are not achievable in tamoxifen treated women, may have more substantial anti-cancer effects and should therefore be explored.

## Materials and Methods

### Cell Culture and Chemicals

MCF7 breast cancer cells were purchased from American Type Culture Collection (Manassas, VA) and were routinely grown in phenol red-free Dulbecco’s modified Eagle’s medium/F12 (DMEM/F12) medium containing 10% (v/v) Fetal Bovine Serum (FBS) and 1% (v/v) antibiotic-antimycotic (AA) solution in a humidified 37°C incubator with 5% CO_2_. Cells were cultured in the same medium containing 10% triple charcoal stripped FBS for 48 hours prior to and during all estrogen and anti-estrogen treatments to deprive the cells of hormone exposure and maximize the effects of these ligands. 17β-estradiol (E_2_), (Z)-tamoxifen and (Z)-4-hydroxy-tamoxifen were purchased from Sigma Aldrich (St. Louis, MO). (Z)-N-desmethyl-tamoxifen was purchased from Toronto Research Chemicals (Ontario, Canada). ICI-182,780 was purchased from Tocris Biosciences Inc. (Baldwin, MO). (Z)-endoxifen was synthesized by Dr. Abdul Fauq (Mayo Clinic, Jacksonville, FL). All ER ligands used in this study were resuspended in 100% ethanol.

### Chromatin Immunoprecipitation (ChIP) Assays

MCF7 cells were plated in 100 mm tissue culture plates at a density of approximately 50% and were transfected with 5 µg of an estrogen response element (ERE) -TK-luciferase reporter construct using Fugene6 (Roche Applied Science, Indianapolis, IN). Following transfection, cells were treated in triplicate as indicated for either 1 or 24 hours and ChIP assays were performed as previously described [22]. Immunoprecipitations were conducted using 0.5 µg of an ERα specific antibody (HC-20, Santa Cruz, CA). Inputs were generated in an identical manner excluding the antibody immunoprecipitation step. Quantitative Real-Time PCR was conducted in triplicate on all samples and a representative data set is shown. Primers used in the PCR reactions were designed to surround the known ERE’s and are listed in Table S6. Quantitative PCR values were calculated based on the threshold cycle (C_t_) and were normalized to input values.

### Cell Treatments and RNA Isolation

For microarray analyses, MCF7 cells were plated at a density of approximately 70% in 100 mm tissue culture dishes. Cells were treated in triplicate with estrogen or anti-estrogens as indicated for 24 hours. Total RNA was isolated using Trizol reagent (Invitrogen). RNA yield was determined using a NanoDrop 1000 spectrophotometer (Thermo Fisher Scientific, Wilmington, DE). RNA integrity and quality were determined by capillary electrophoresis on an Agilent 2100 Bioanalyzer (Agilent Technologies, Santa Clara, CA).

### Illumina Microarray Analysis

Changes in gene expression profiles were determined using Illumina HumanHT-12 expression BeadChips to screen more than 27,000 annotated genes represented by 48,804 probes by Mayo Clinic’s Advanced Genomics Technology Center (Rochester, MN). Data was processed using BeadStudio Version 3.1 and normalized using the fastlo function [23] implemented in the statistical package R. Data were filtered to exclude probes (referred to as genes throughout) whose expression was at or below background levels as determined by detection P-values (≥0.05). Pair-wise comparisons were made to identify differentially expressed genes using LIMMA. Genes were determined to be significantly regulated if their differential P-value was <0.05. Fold-changes were calculated by raising 2 to the power of the mean difference (log 2 scale) between the treatment groups and controls. The microarray data presented here is available in GEO (http://www.ncbi.nlm.nih.gov/geo/) under accession #: GSE43702.

The heat maps presented in the figures throughout this manuscript were generated using only those genes that were differentially expressed in at least one of the treatment groups included in the comparison and which had average fold changes >3 standard deviations from all other genes in the comparison. Each heat map depicts a standardized average fold change for each gene across triplicate experiments. The heat maps presented in the supplemental figures include the same genes, but depict their standardized relative expression levels in each individual replicate experiment.

### Biological Pathway Analysis

Genes determined to be significantly regulated by a given treatment were further analyzed using MetaCore software (http://www.genego.com/metacore.php) to determine if specific biological pathways were significantly enriched within a given treatment. More specifically, genes with differential expression P-values <0.05 and fold-changes >1.5 from each comparison were used as focus genes and a hypergeometric test was applied to each of over 600 canonical pathways. Pathways with P-values <0.05 suggested a significant enrichment. To adjust for multiple testing, a false discovery rate of 0.25 was used.

### Real-Time Reverse Transcription Polymerase Chain Reaction

Five hundred ng of total RNA was reverse transcribed using the iScript™ cDNA Synthesis Kit (Bio-Rad). Real-time PCR was performed in triplicate using a Bio-Rad iCycler (Hercules, CA) and a PerfeCTa™ SYBR Green Fast Mix™ for iQ real-time PCR kit (Quanta Biosciences, Gaithersburg, MD) as specified by the manufacturer. Cycling conditions were as follows: 95°C for 2 minutes followed by 40 cycles of 95°C for 5 seconds and 60°C for 30 seconds. Melt curves were generated to ensure amplification of a single PCR product. Quantitation of the PCR results were calculated based on the threshold cycle (C_t_) and were normalized using TATA Binding Protein as a control. All PCR primers were designed using Primer3 software (http://frodo.wi.mit.edu/primer3/) and were purchased from Integrated DNA Technologies (Coralville, IA). Primer sequences are listed in Table S6.

### Cell Cycle Analysis

MCF7 cells were cultured in 3x-charcoal stripped serum containing media for 48 hours prior to plating at a density of approximately 70% in 100 mm tissue culture dishes in the same media. Cells were treated in triplicate as indicated for 24 hours. One million cells from each treatment were washed in 1X PBS and fixed in 95% ethanol for 3 minutes at room temperature. Cells were washed again with PBS, resuspended in 1 ml of PBS containing 0.2 mg/ml RNase A and incubated in a 37°C water bath for 30 minutes. Cells were again washed with PBS and subsequently incubated in 1 ml of PBS containing 20 µg/ml Propidium Iodide in the dark on ice. Cell cycle distribution was analyzed by flow cytometry (FACSCalibur) by the Mayo Clinic Flow Cytometry Core Facility. The percentage of cells in G1, S and G2/M phase were determined for each treatment and averaged across triplicate experiments.

## Supporting Information

Figure S1 **Heat map analysis of genes regulated by estrogen +100 nM levels of 4HT, endoxifen or ICI.** Heat maps were generated using hierarchical clustering of genes that were differentially expressed in at least one of the indicated treatment groups relative to vehicle control and which had average fold-changes >3 standard deviations from all other genes in the comparison. The relative expression levels for each gene are shown across all individual treatment replicates. Red indicates increased gene expression while green indicates decreased gene expression relative to vehicle treated controls.
(TIF)

Figure S2 **Heat map analysis of genes regulated by 100 nM levels of 4HT, endoxifen or ICI.** Heat maps were generated using hierarchical clustering of genes that were differentially expressed in at least one of the indicated treatment groups relative to vehicle control and which had average fold-changes >3 standard deviations from all other genes in the comparison. The relative expression levels for each gene are shown across all individual treatment replicates. Red indicates increased gene expression while green indicates decreased gene expression relative to vehicle treated controls.
(TIF)

Figure S3 **Heat map analysis of endoxifen concentration dependent gene expression changes.** Heat maps were generated using hierarchical clustering of genes that were differentially expressed in at least one of the indicated treatment groups relative to E_2_, TAM, 4HT and NDT treated cells and which had average fold-changes >3 standard deviations from all other genes in the comparison. The relative expression levels for each gene are shown across all individual treatment replicates. Red indicates increased gene expression while green indicates decreased gene expression relative to vehicle treated controls.
(TIF)

Table S1 **Genes determined to be significantly regulated by 24 hour treatments with E2 (10 nM) alone, or in combination with 100 nM concentrations of 4HT, endoxifen or ICI relative to vehicle controls.**
(XLS)

Table S2 **Genes determined to be significantly altered by the addition of an anti-estrogen+estrogen relative to estrogen treatment alone.**
(XLS)

Table S3 **Genes determined to be significantly regulated by estrogen +4HT, estrogen+endoxifen or estrogen+ICI, but not by estrogen treatment alone.**
(XLS)

Table S4 **Genes determined to be significantly regulated by 24 hour treatments with 100 nM concentrations of 4HT, endoxifen or ICI relative to vehicle controls.**
(XLS)

Table S5 **Genes determined to be significantly regulated by the addition of various endoxifen concentrations relative to cells treated with estrogen (10 nM), tamoxifen (300 nM), NDT (700 nM) and 4HT (7 nM) alone.**
(XLS)

Table S6 **Information on primers used in this manuscript.**
(XLS)
