# Supplementary figures and images for: Endoxifen’s Molecular Mechanisms of Action Are Concentration Dependent and Different than That of Other Anti-Estrogens
Source: PLoS One. 2013 Jan 28;8(1):e54613. doi: 10.1371/journal.pone.0054613 (PMC3557294; doi:10.1371/journal.pone.0054613)

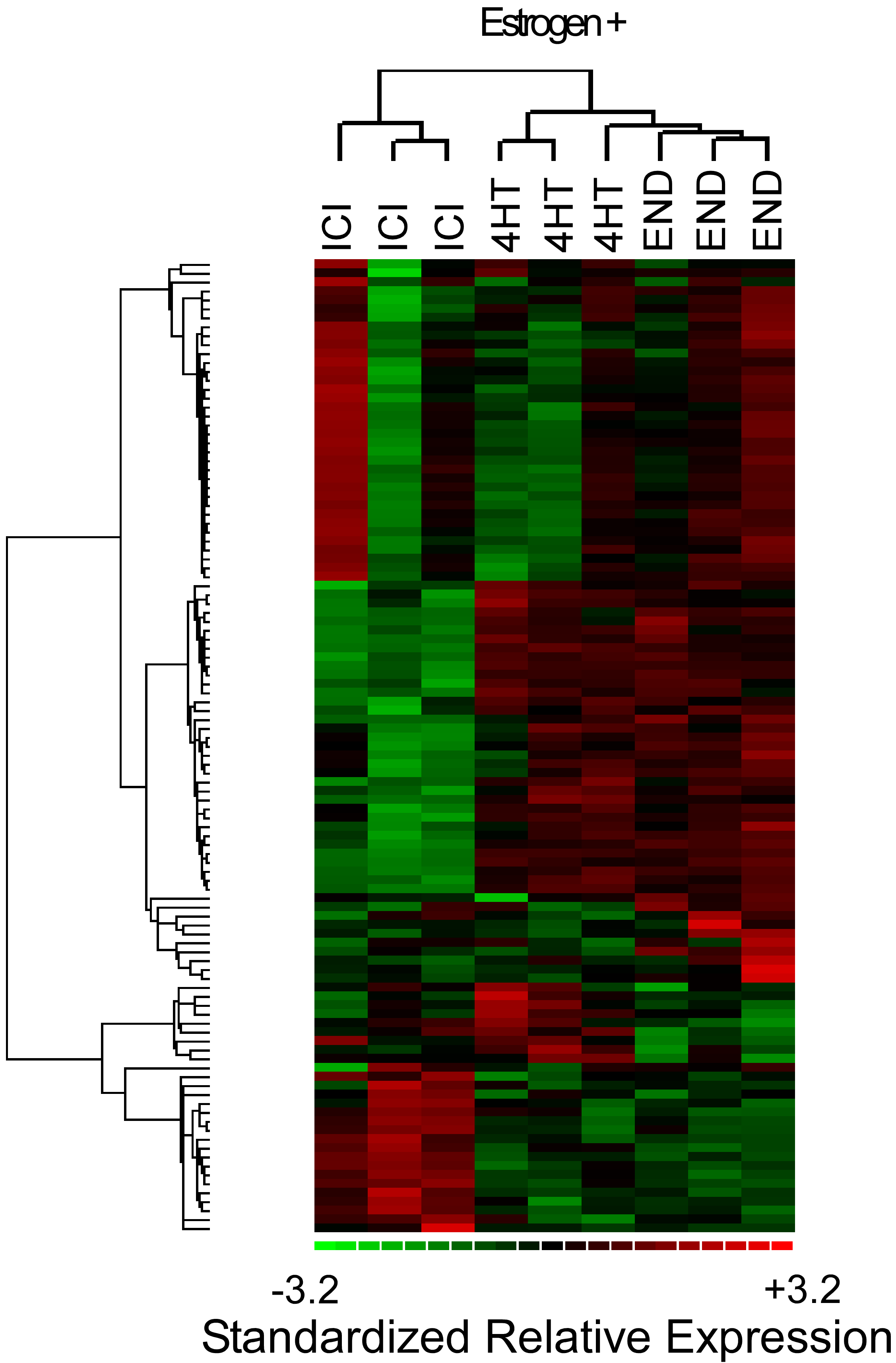

Supplement: Figure S1 — Heat map analysis of genes regulated by estrogen +100 nM levels of 4HT, endoxifen or ICI. Heat maps were generated using hierarchical clustering of genes that were differentially expressed in at least one of the indicated treatment groups relative to vehicle control and which had average fold-changes >3 standard deviations from all other genes in the comparison. The relative expression levels for each gene are shown across all individual treatment replicates. Red indicates increased gene expression while green indicates decreased gene expression relative to vehicle treated controls. (TIF) [file pone.0054613.s001.tif]

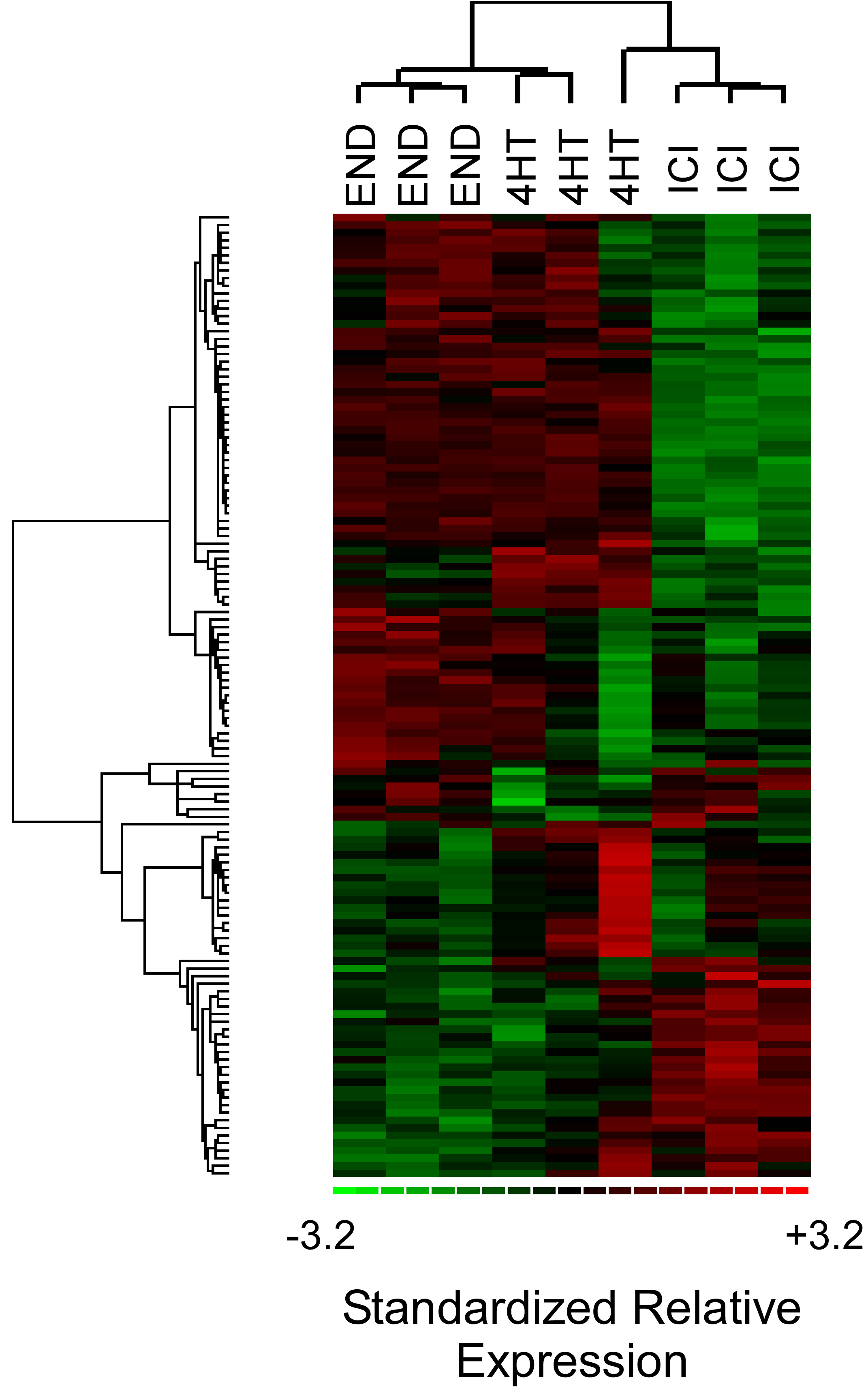

Supplement: Figure S2 — Heat map analysis of genes regulated by 100 nM levels of 4HT, endoxifen or ICI. Heat maps were generated using hierarchical clustering of genes that were differentially expressed in at least one of the indicated treatment groups relative to vehicle control and which had average fold-changes >3 standard deviations from all other genes in the comparison. The relative expression levels for each gene are shown across all individual treatment replicates. Red indicates increased gene expression while green indicates decreased gene expression relative to vehicle treated controls. (TIF) [file pone.0054613.s002.tif]

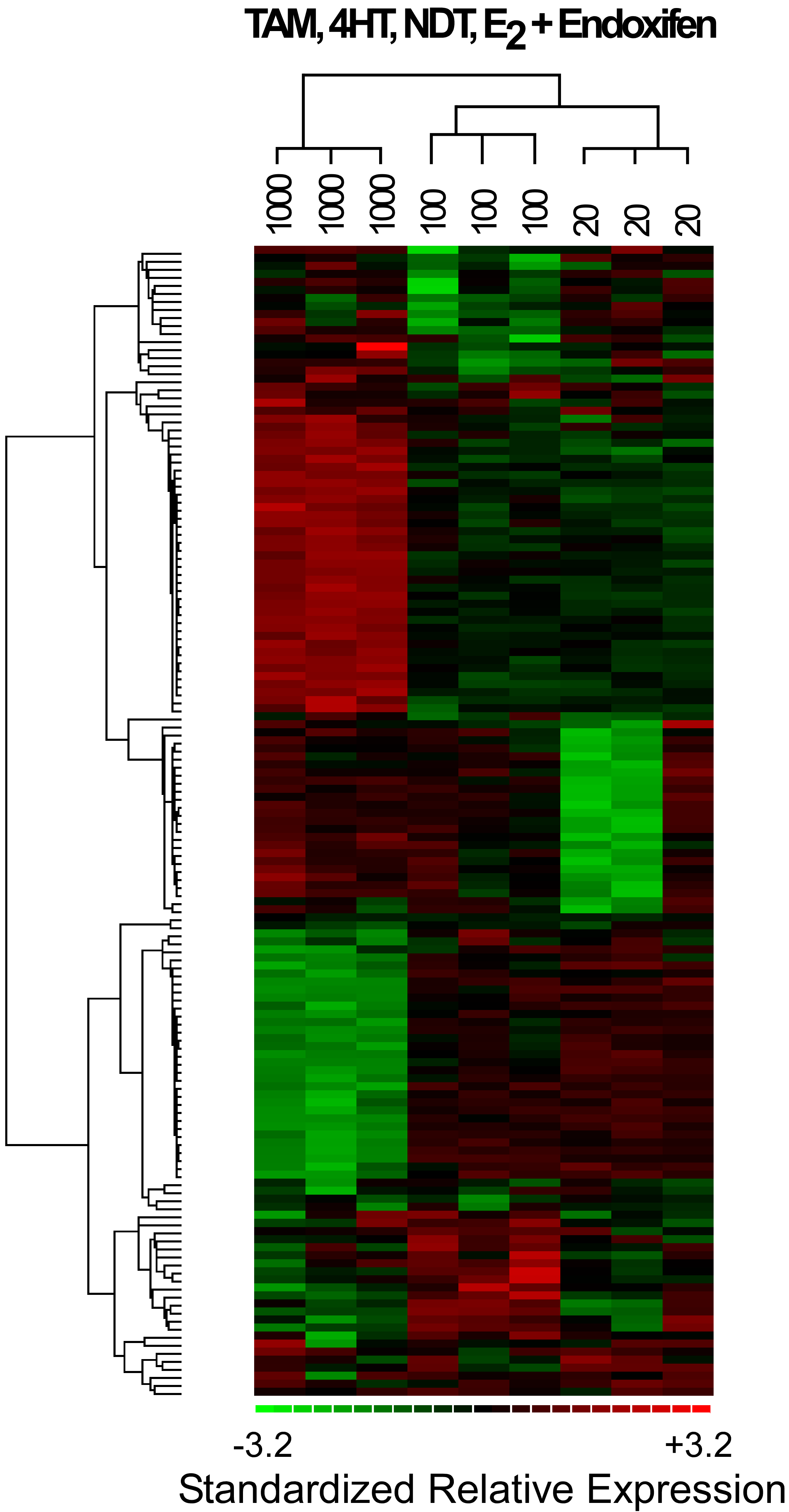

Supplement: Figure S3 — Heat map analysis of endoxifen concentration dependent gene expression changes. Heat maps were generated using hierarchical clustering of genes that were differentially expressed in at least one of the indicated treatment groups relative to E2, TAM, 4HT and NDT treated cells and which had average fold-changes >3 standard deviations from all other genes in the comparison. The relative expression levels for each gene are shown across all individual treatment replicates. Red indicates increased gene expression while green indicates decreased gene expression relative to vehicle treated controls. (TIF) [file pone.0054613.s003.tif]
